# Adipocytes and Obesity-Related Conditions Jointly Promote Breast Cancer Cell Growth and Motility: Associations With CAP1 for Prognosis

**DOI:** 10.3389/fendo.2018.00689

**Published:** 2018-11-22

**Authors:** Ann H. Rosendahl, Malin Bergqvist, Barbara Lettiero, Siker Kimbung, Signe Borgquist

**Affiliations:** ^1^Division of Oncology and Pathology, Department of Clinical Sciences Lund, Lund University and Skåne University Hospital, Lund, Sweden; ^2^Departments of Clinical Medicine/Oncology, Aarhus University and Aarhus University Hospital, Aarhus, Denmark

**Keywords:** breast cancer, obesity, adipocytes, CAP1, metabolic conditions, prognosis

## Abstract

The global increase in overweight and obesity rates represent pressing public health concerns associated with severe comorbidities, amongst a rising incidence and impaired outcome of breast cancer. Yet, biological explanations for how obesity affects breast cancer are incompletely mapped. Herein, the joint impact by differentiated 3T3-L1 adipocytes and obesity-related metabolic conditions on breast cancer cells was evaluated *in vitro* and adipocyte-derived mediators assessed. Adipokine receptor expression was explored among breast cancer cell lines (*n* = 47) and primary breast tumors (*n* = 1,881), where associations with survival outcomes were investigated. Adipocytes and metabolic complications jointly stimulated breast cancer cell proliferation and motility, with phenotype-specific differences. Resistin was among the top modulated adipokines secreted by 3T3-L1 adipocytes under obesity-associated metabolic conditions compared with normal physiology. The newly identified resistin receptor, CAP1, was expressed across a large panel of breast cancer cell lines and primary breast tumors. *CAP1* was associated with poor tumor characteristics with higher *CAP1* expression among estrogen receptor (ER)-negative tumors, relative to ER-positive tumors (*P* = 0.025), and higher histological grades (*P* = 0.016). High *CAP1* tumor expression was associated with shorter overall survival (adjusted hazard ratio [HR_adj_] 1.54; 95% confidence interval [CI], 1.11–2.13) and relapse-free survival (HR_adj_ 1.47; 95% CI, 1.10–1.96), compared with low or intermediate *CAP1* expression, particularly among ER-positive tumors or lymph node positive tumors. Together, these translational data demonstrate that the adipocyte secretome promote breast cancer cell proliferation and motility and highlight a potential role of CAP1 regarding breast cancer outcome—results that warrant further investigation to elucidate the obesity-breast cancer link in human pathology.

## Introduction

As a consequence of changing diet and lifestyle patterns over the last decades, overweight and obesity are pressing global health concerns with rapidly escalating rates. According to the current estimates by the World Health Organization, almost 2 billion adults worldwide (39%) are overweight or obese (1). Increased body fatness poses a major risk for developing several types of cancer, including breast cancer (2, 3). Obesity is additionally associated with advanced disease stage and impaired prognosis after breast cancer onset (3). Although obesity has substantial impact on breast cancer, the relationship is complex and not fully understood.

Overweight and obese individuals frequently acquire metabolic complications where gradually increasing defects in insulin signaling result in chronic hyperinsulinemia (pre-type 2 diabetes; pre-T2D), insulin resistance (overt-T2D), and impaired insulin secretion (late-T2D). These conditions are associated with hyperglycemia and increased insulin and insulin-like growth factor 1 (IGF1) levels that may contribute to breast cancer initiation, progression and resistance to treatment (4, 5). Along with these systemic influences of obesity, it is increasingly recognized that a dynamic communication between the different cell types and extracellular matrix within the tumor microenvironment is an integral part of breast cancer development. Due to their proximity in the mammary gland tissue, adipocytes are considered to play a role in breast cancer development and progression (6). In breast cancer, early local invasion occurs in close proximity of adipocytes at the invasive front (7). Still, the cellular and molecular interactions between adipocytes and breast epithelial cells, along with the role of adipocytes and obesity-associated metabolic conditions in tumor progression remain incompletely understood.

Adipose tissue has historically been considered to merely be an energy depot. More recently it is described as an endocrine organ producing several biologically active factors, collectively termed adipokines, including enzymes, hormones, growth factors, and inflammatory cytokines, both at the systemic level and locally in the breast (5). The increased risk of estrogen-responsive postmenopausal breast cancer among obese women may in part relate to the higher levels of circulating and local estrogen from the excess adipose tissue (5). However, independent of menopausal status, tumor stage and hormone receptor status, obesity has been suggested to be a negative prognostic factor for breast cancer. An increase in lymph node involvement and a higher propensity to distant metastasis has also been established in obese women (3, 8). Consequently additional obesity-induced mediators, beyond elevated estrogen levels, likely play a significant role in disease progression and impaired prognosis among obese breast cancer patients.

Resistin is a novel adipokine identified that suppresses glucose tolerance and insulin sensitivity, and has been proposed to be an important link between obesity, insulin resistance and type 2 diabetes (T2D) (9). Resistin, for resistance to insulin, was initially described to be exclusively secreted by adipocytes, although more recent reports demonstrate that monocytes and macrophages associated with the chronic low-grade inflammation in obese humans also secrete this peptide (10). Several studies have reported upregulated systemic resistin levels in breast cancer patients, while others have not shown the same correlation (11–14). As resistin, like other adipokines is present circulating in the blood, it may act on breast cancer both via its systemic endocrine effects, as well as locally in the tumor microenvironment through paracrine actions. Studies of the biological properties and function of resistin have been hampered by the unknown identity of its cognate signaling receptor. First in 2014, it was demonstrated that resistin binds and acts through CAP1 (Adenylate Cyclase-Associated Protein 1), and the *bona fide* receptor for resistin was revealed (15). Yet, the impact by resistin and in particular CAP1 on breast cancer remains elusive.

The objective of this study was to expand the clinical observations on obesity and breast cancer to explore the joint influence by adipocytes and obesity-associated metabolic conditions on local cellular features with impact on breast cancer progression and migratory capabilities. An additional aim was to identify obesity-associated adipokines putatively involved in mediating the cellular effects and to evaluate the corresponding adipokine receptor expression levels in primary breast tumors in relation to clinical breast cancer outcome.

## Materials and methods

### Reagents

All chemicals and reagents, including the anti-CAP1 (HPA030124) and anti-glyceraldehyde-3-phosphate dehydrogenase (GAPDH, MAB374) antibodies, were purchased from Sigma Aldrich unless stated otherwise. Cell culture media, penicillin/streptomycin, insulin, NuPAGE gels and MOPS buffer were purchased from Invitrogen. Bovine calf serum was purchased from ATCC-LGC Standards. PBS was from HyClone.

### Cell culture

The ERα-positive human breast cancer cell lines T47D (non/low invasive), MCF-7 (low invasive), the triple-negative MDA-MB-231 (highly invasive), and the pre-adipocyte 3T3-L1 fibroblast cell line were purchased from and validated by ATCC-LGC Standards. The cells were maintained in Dulbecco's Modified Eagle's Medium (DMEM) supplemented with antibiotics (100 U/mL penicillin and 100 μg/mL streptomycin) and 10% fetal bovine serum (breast cancer cells) or 10% bovine calf serum (3T3-L1) in a humidified 5% CO_2_ atmosphere at 37°C, and routinely used below passage 35. For *in vitro* adipocyte differentiation, 3T3-L1 pre-adipocytes cells were grown to confluence in growth media followed by 48 h exposure to adipocyte differentiation medium [DMEM supplemented with FBS (10%), dexamethasone (1.0 μmol/L), methylisobutylxanthine (IBMX; 0.5 mmol/L), insulin (1.0 μg/mL) and antibiotics as above]. The 3T3-L1 adipocytes were subsequently maintained in adipocyte maintenance medium [AMM; DMEM supplemented with 10% FBS, insulin (1.0 μg/mL) and antibiotics as above] and full differentiation obtained within 7–14 days.

### Oil red-O staining

**Figure 1:**
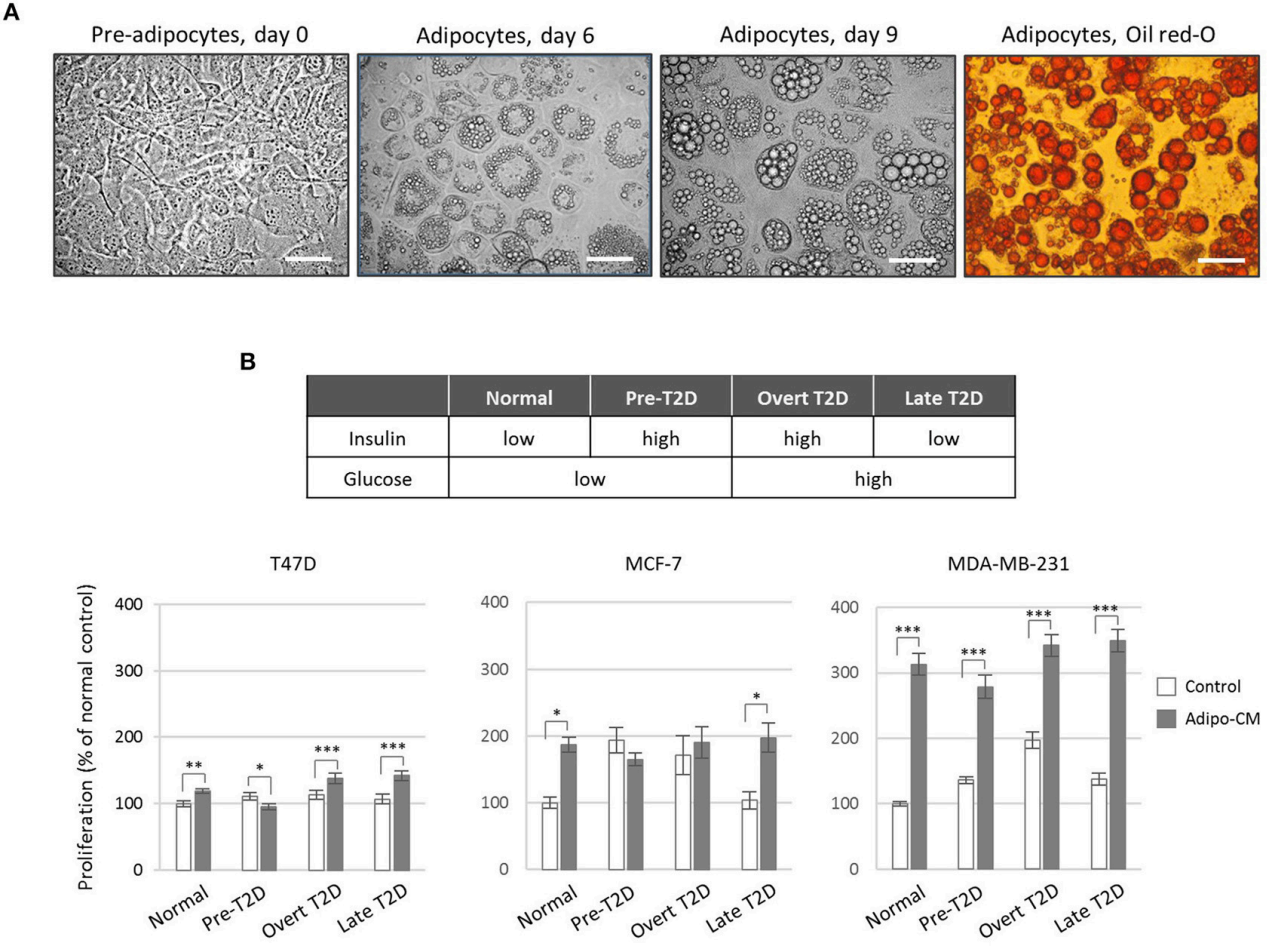
Effect by the adipocyte secretome on proliferation of human breast cancer cells**. (A)** Photomicrograph images of *in vitro* cultured 3T3-L1 pre-adipocytes and differentiated adipocytes at x40 original magnification. Scale bars indicate 50 μm. Adipocyte differentiation was validated by Oil-Red O staining. **(B)** Proliferation of human breast cancer cells in response to adipocyte-secreted factors. Human T47D, MCF-7 and MDA-MB-231 breast cancer cells were cultured in the presence of adipocyte conditioned media (gray bars) or control (white bars) under normal, pre-T2D, overt-T2D, or late-T2D conditions for 72 h. Results are shown as percentage proliferation of normal control and represent mean ± SE of three independent experiments, each performed with five replicates. *P*-values are based on two-way ANOVA with Sidak's multiple comparisons test. ^*^P < 0.05, ^**^P < 0.01, ^***^P < 0.001.

Adipocyte differentiation was verified using Oil Red-O staining of cytoplasmic accumulation of neutral triglycerides and cholesteryl oleate lipids (Figure 1A). Briefly, adipocytes were fixed in 3% paraformaldehyde followed by Oil Red-O staining. Intracellular lipid droplets were examined by light microscopy at 40x magnification.

### Obesity-related metabolic conditions and adipocyte conditioned media

Differentiated adipocytes were cultured for 24 h under various metabolic conditions in serum-free media [SFM; DMEM supplemented with low glucose (5 mmol/L) or high glucose (25 mmol/L), low insulin (0.1 ng/mL) or high insulin (1.0 μg/mL), BSA (0.2 mg/mL), sodium bicarbonate (1.2 mg/mL), transferrin (0.01 mg/mL) and antibiotics as above] in order to mimic normal physiology (low glucose/low insulin) or the obesity-related metabolic conditions pre-T2D (chronic hyperinsulinemia; low glucose/high insulin), overt-T2D (insulin resistance; high glucose/high insulin) or late-T2D (impaired insulin secretion; high glucose/low insulin). Adipocyte secretome-containing conditioned media (adipo-CM) was subsequently collected, any residual cell debris were removed by centrifugation and adipo-CM aliquots were stored at −20°C until further use. Equivalent SFM media and metabolic conditions, in the absence of adipocytes, were used as controls.

### Cell proliferation assay

T47D, MCF-7 and MDA-MB-231 cells (3, 2.5, and 2 × 10^3^ cells/well, respectively) were seeded in 96-well plates in growth medium for 24 h, washed in PBS and then exposed to adipo-CM or control media as indicated for 72 h. Cell proliferation was subsequently determined using the sulforhodamine B (SRB) assay as a measure of total cell density, as described in (16). Briefly, cells were fixed in ice-cold 17% trichloroacetic acid, stained with 0.4% (w/v) SRB and unbound SRB removed by washing in 1% acetic acid. Protein-bound SRB was dissolved using 10 mM TRIS base buffer and quantified on a VersaMax microplate reader at 570 nm using the SoftMax Pro software (Molecular Devices).

### Morphology and cellular protrusions

The three breast cancer cell lines were exposed to adipo-CM or control medium for 48 h where after morphology and cellular protrusions were evaluated. Cell micrographs were captured at 10x magnifications with Nikon Eclipse TE200 microscope using Zeiss AxioCam ERc 5 s camera and Axio Vision Rel 4.8 software. Total number of cellular protrusions were quantified for T47D cells within nine random microscopic fields using Image J software (NIH, USA) and normalized against the corresponding total cell numbers. For each experiment, a minimum of 1,000 cells were quantified per condition. F-actin staining by Alexa Fluor 488 Phalloidin was performed according to the manufacturer's instructions (ThermoFisherScientific).

### Cell migration

Cell migration was measured using the *in vitro* scratch assay, as previously described (17). Briefly, breast cancer cells were grown to confluence and subsequently starved for 24 h in SFM. An artificial gap (cross wound) was created in the cell monolayer, and the cells were washed twice with PBS to remove cellular debris. The cells were then incubated with adipo-CM or control medium under the metabolic conditions described above, and migration photographed with Nikon Eclipse TE200 microscope and Zeiss AxioCam ERc 5 s camera at 10x magnification at regular time intervals (0–48 h). Percent gap closure compared with time 0 h was quantified as a measure of cell migration using TScratch software (Computational Science & Engineering Laboratory, ETH Zürich, Switzerland) (18).

### Proteome profiler array

Secreted adipocyte-derived mediators were assessed in adipo-CM from normal physiology and obesity-related metabolic conditions using the Proteome Profiler™ Human Adipokine Array, according to the manufacturer's instructions (R&D Systems). Relative levels of secreted adipokines were quantified by analyzing the average pixel density after background correction using ImageJ software (NIH). Heat map of fold difference in relative adipokine abundance compared with normal was generated by comparing the pixel densities (normalized to internal assay controls) of the corresponding signals.

### Western immunoblotting

The breast cancer cell lysates were prepared using RIPA buffer with protease and phosphatase inhibitors and protein concentration determined, as described previously (19). Equal amounts of protein samples (20 μg) were separated by pre-cast SDS-PAGE (NuPAGE 10% Bis-Tris, Invitrogen) and transferred to nitrocellulose membranes. The membranes were blocked with 5% (w/v) milk in Tris-buffered saline Tween-20 (TBST) and probed with antibodies to CAP1 (1:750) or GAPDH (1:1000) as a loading control. Protein abundance was detected with horseradish peroxidase specific secondary antibodies and visualized by SuperSignal West Extended Duration Substrate (ThermoFisherScientific) using the Alpha Innotech FluorChem® FC2 imaging system and AlphaView version 3.0.3.0 software (ProteinSimple) or ImageJ software.

### *CAP1* mRNA expression

*CAP1* mRNA expression was examined in a panel of 47 human breast cancer cell lines and 1,881 primary breast tumors (representing the cellular heterogeneity within the tumor microenvironment) processed as previously described (20, 21). The cell lines were classified into clinical subtypes as specified in Neve et al. (22), and the primary breast tumors according to PAM50 subtypes (20). The median age among the patients was 55 ± 13 years, with an average tumor size of 20 ± 12 mm and 75% estrogen receptor positive tumors. Independent correlation analyses of resistin *RETN* and *CAP1* mRNA expression and protein levels (using paired RNASeqV2 and mass spectrometry data, respectively), co-expressed genes and biological network analysis were conducted in a subset of 1,105 invasive breast carcinomas within The Cancer Genome Atlas (TCGA) project.

### Statistical analysis

*In vitro* experimental data are expressed as mean ± SE of three independent experiments and treatment groups were compared using two-way ANOVA, followed by Sidak's or Bonferroni's multiple comparisons tests. Overall survival (OS) and relapse-free survival (RFS) were estimated using the Kaplan-Meier method. Univariable survival analyses of breast cancer outcomes in relation to *CAP1* expression were performed using the log-rank test. The impact of *CAP1* expression on prognosis was additionally analyzed using multivariable Cox regression analyses to calculate hazard ratios (HR) with 95% confidence intervals (CI), adjusted for potential confounding factors; age (≤50 years or >50 years), tumor size (≤20 mm or >20 mm), lymph node (LN) status (negative or positive), histologic grade (1–2 or 3) and estrogen receptor (ER) status (positive or negative). Pearson or Spearman correlation coefficients were used for correlation assessments, as indicated. Not all samples had clinical information for all covariates or clinical follow-up, and the number of patients included in the different analyses are shown as (*n*). The REporting recommendations for tumor MARKer prognostic studies (REMARK) guidelines were followed (23). GOBO: Gene expression-based Outcome for Breast cancer Online (20, 21) and cBioPortal (24, 25) platforms, as well as IBM SPSS Statistics 23, GraphPad Prism 7 and Excel 2007 softwares were used for data and statistical analyses. A *P*-value of < 0.05 was considered statistically significant.

## Results

### Adipocyte secretome promote breast cancer cell proliferation

To investigate the joint influence by adipocytes and obesity-associated metabolic alterations on cell proliferation, human breast cancer cells of different phenotypes (26, 27) were exposed to adipocyte secretome-containing conditioned media (adipo-CM) from successfully differentiated adipocytes (Figure 1A) cultured under various metabolic conditions (Figure 1B). Exposure to adipocyte-secreted factors significantly stimulated cell proliferation of all three cell lines investigated (by 19% to 213%) compared with control media under normal metabolic conditions (Figure 1B). The two estrogen receptor (ER)-positive, luminal-like T47D and MCF-7 cells were low- and intermediate responsive, respectively, while the triple-negative MDA-MB-231 cells were highly responsive (Figure 1B). The joint influence by adipocyte-derived factors and metabolic pressure mimicking overt or late T2D conditions, further stimulated the proliferation of both T47D and MDA-MB-231 cells to a higher magnitude (maximum increase by 42 and 250%, respectively), while no additional stimulation was observed for MCF-7 cells.

### Increased abundance of stellate protrusions by non-invasive T47D cells in response to combined adipo-CM and obesity-associated metabolic pressures

**Figure 2:**
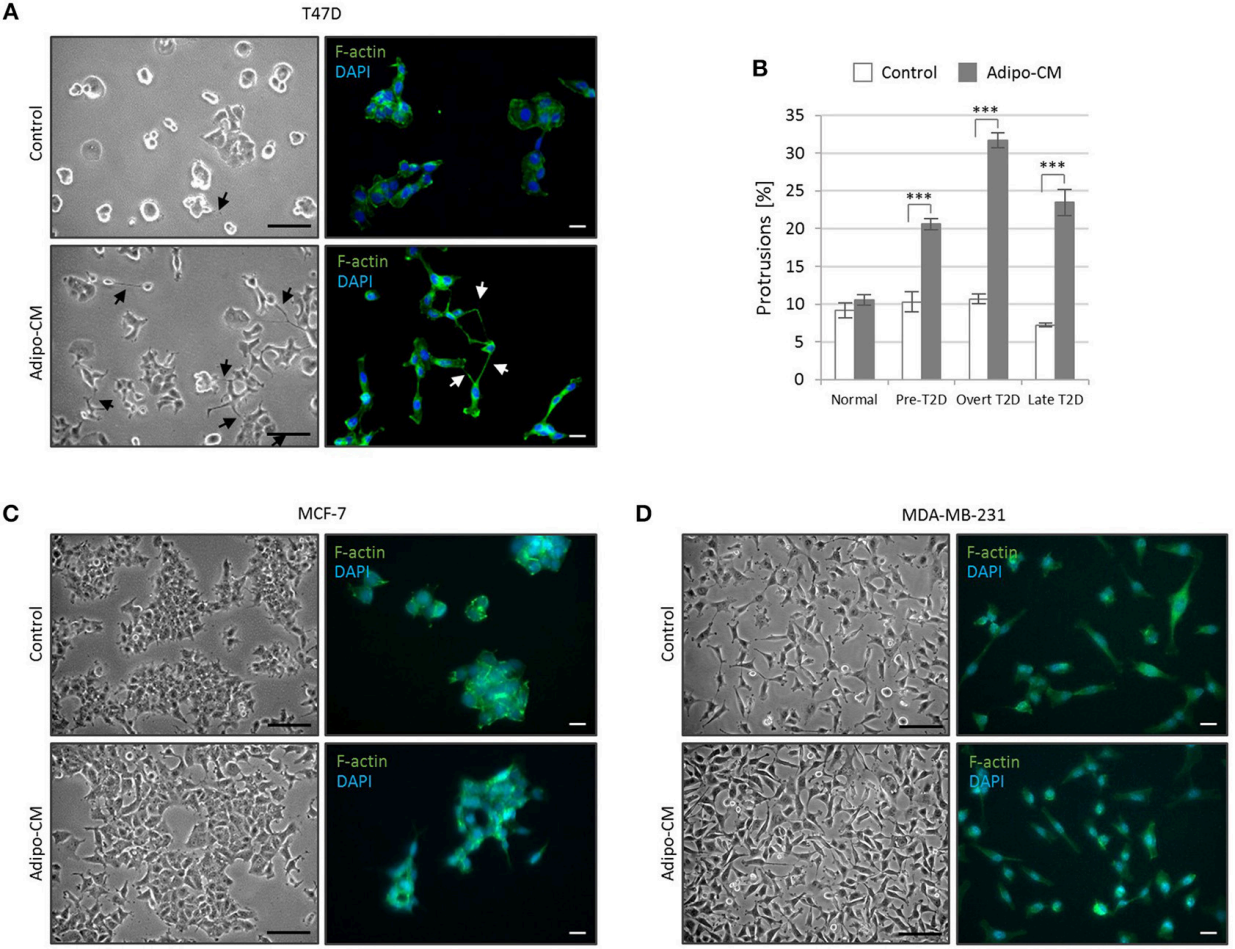
Adipocyte-derived factors stimulate morphology alterations and protrusion extensions in T47D cells. The non-/low-invasive T47D cells were exposed to adipocyte conditioned media or control media under normal, pre-T2D, overt-T2D or late-T2D conditions for 48 h. **(A)** Representative bright field images and immunofluorescence cytoskeletal F-actin staining depicting cell morphology of T47D cells in control or adipo-CM under overt T2D conditions. Arrows indicate cellular protusions. Scale bars: 50 and 20 μm, respectively. **(B)** Graph shows quantification of percent cellular protrusions relative to total T47D cell numbers after exposure to adipocyte-derived factors (gray bars) or control (white bars). Data is presented as mean ±SE from nine random microscopic fields from one representative of three independent experiments. A minimum of 1,000 cells were quantified per condition and experiment. *P*-values based on two-way ANOVA with Bonferroni correction displaying significant differences between cells exposed to adipo-CM and control. ^***^P < 0.001. **(C,D)** Images showing cell morphology and F-actin staining of MCF-7 and MDA-MB-231 cells in control or adipo-CM under overt T2D conditions. Scale bar 50 and 20 μm, respectively.

While the epithelial-like T47D cells showed weak proliferative response to adipo-CM, they displayed distinct morphological alterations and cytoskeletal F-actin rearrangements in response to adipocyte-derived factors combined with obesity-related metabolic conditions (Figure 2A). Under normal control growth conditions, the non-invasive T47D cells formed colonies with tightly cohesive mass structures (Figure 2A). Following exposure to adipo-CM under obesity-associated metabolic conditions, the T47D cells acquired an elongated cell shape with stellate projections, indicative of a more motile phenotype. No marked difference in membrane protrusions were observed in response to adipo-CM relative to control under normal metabolic conditions. Quantification revealed a significant adipo-CM stimulated increase in protrusions under pre-, overt or late T2D conditions, compared with control (*P* < 0.001; Figure 2B). No obvious alterations in stellate projections were observed for MCF-7 or MDA-MB-231 cells in response to adipo-CM (Figures 2C,D).

### Adipocyte-derived factors significantly enhance breast cancer cell migration

**Figure 3:**
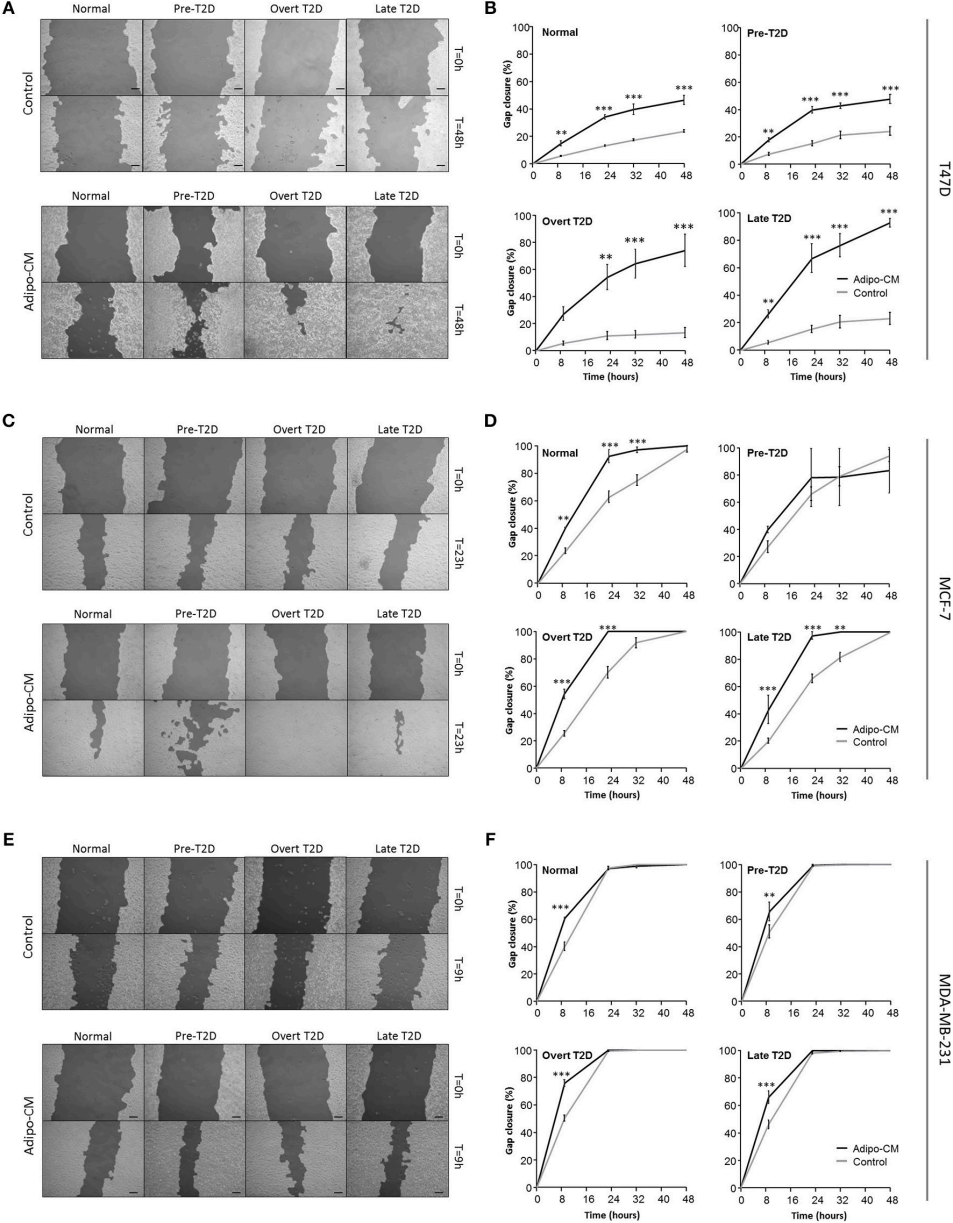
Adipocyte-derived mediators stimulate cell migration. Effects by adipocyte-derived factors and obesity-associated metabolic conditions on breast cancer cell migration were analyzed by scratch assay and automated quantification. **(A,C**,**E)** Representative images of cell migration in control or adipo-CM at the indicated time points for **(A)** T47D, **(C)** MCF-7, and **(E)** MDA-MB-231 cells. The lighter gray area show cell monolayers, and darker area show open scratch. Scale bar: 100 μm. **(B**,**D**,**F)** Graphs show quantification of percent gap closure as a measure of migration over time compared with time 0 for **(B)** T47D, **(D)** MCF-7, and **(F)** MDA-MB-231 breast cancer cells exposed to adipo-CM or control and the indicated metabolic conditions. Results are presented as mean ± SE (*n* = 3–4) from one representative of three experiment. Statistical analysis was performed by two-way ANOVA with Bonferroni correction; significant differences between cells exposed to adipo-CM and control shown as ^**^P < 0.01 and ^***^ *P* < 0.001.

Following the observed cellular and morphological alterations indicative of a more motile phenotype, the joint influence by adipocyte-derived factors and obesity-associated metabolic conditions on breast cancer cell migration was evaluated next. Exposure to adipo-CM significantly enhanced the migration of the inherent non-/low invasive T47D cells, compared with control. Under normal or pre-T2D conditions, the migration was induced 1-fold in response to adipo-CM, compared with control (*P* < 0.001; Figures 3A,B). The effect was even further enhanced under overt- or late T2D conditions reaching a 4-fold and 3-fold increase, respectively, at 48 h compared with control (*P* < 0.001; Figures 3A,B). Similarly, the migration of MCF-7 cells was enhanced in the presence of adipo-CM under normal, overt or late T2D conditions (*P* < 0.01; Figures 3C,D). For the highly invasive MDA-MB-231 cells, the magnitude of migration was increased in response to adipo-CM at the early 9-h time point for all metabolic conditions, compared with controls (*P* < 0.01; Figures 3E,F). However, by 24 h both adipo-CM-stimulated MDA-MB-231 cells and controls reached a maximum 100% migration.

### Increased adipokine resistin levels under obesity-associated metabolic conditions

**Figure 4:**
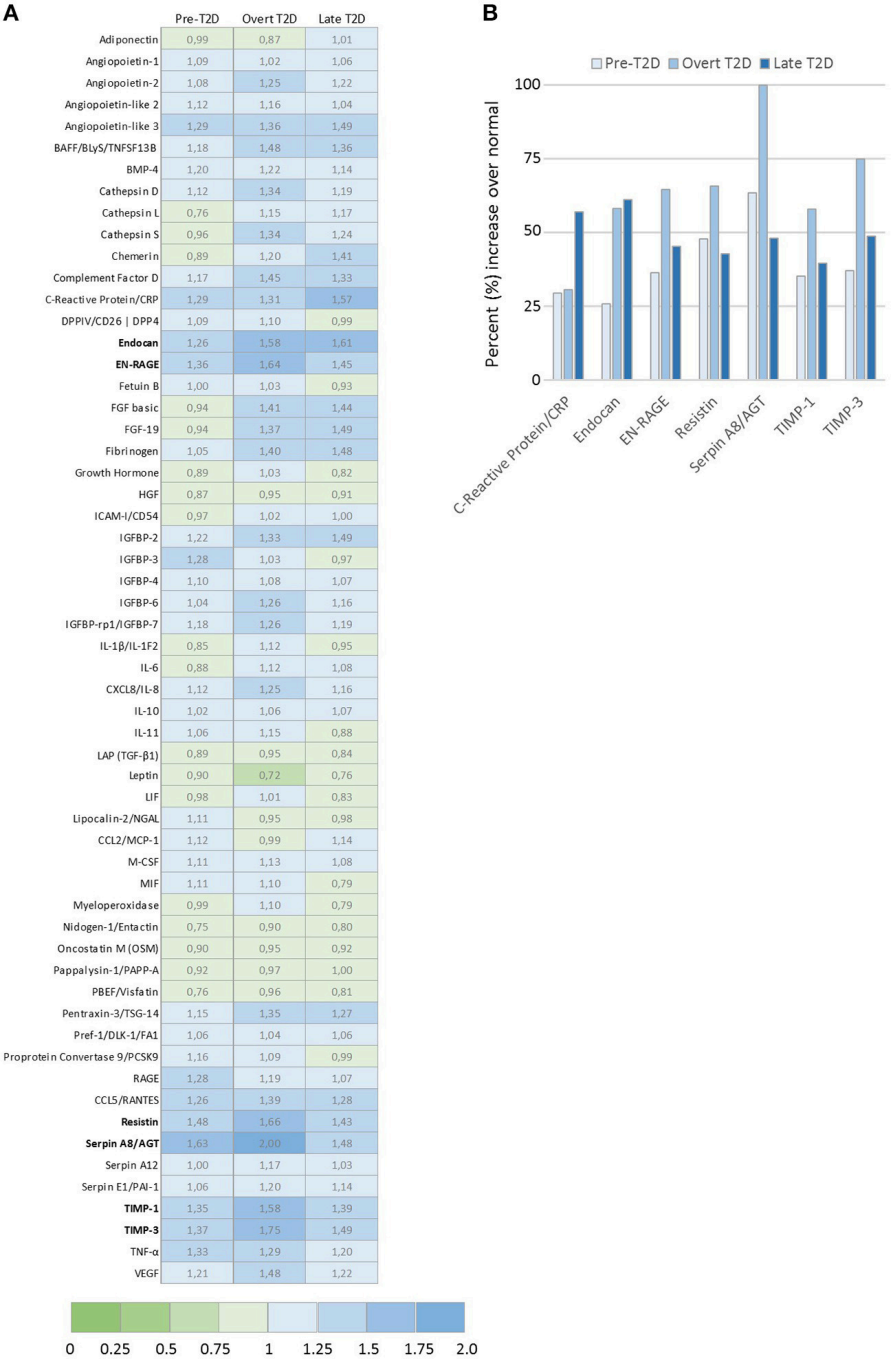
Adipokine proteome profile analysis of relative levels of 58 secreted adipokines by 3T3-L1 adipocytes. **(A)** Graph shows the top modulated adipokines by 3T3-L1 adipocytes cultured under obesity-associated metabolic conditions Pre-T2D, overt T2D, or late T2D, with ≥ 50% increase relative to normal (low glucose/low insulin) metabolic conditions. **(B)** Ratio of relative expression levels of the 58 individual adipokines secreted by 3T3-L1 adipocytes cultured under pre-T2D, overt T2D or late T2D metabolic conditions compared with normal. Relative adipokine levels in normal metabolic conditions = 1, increased levels (>1.0; blue), decreased levels (<1.0; green) compared with normal.

Toward identifying the profiles of secreted adipocyte-derived factors that may contribute to the biological effects detected in the breast cancer cells, relative levels of 58 adipokines were evaluated by proteome profiler arrays (Figure 4A). A panel of the top differentially expressed adipokines was identified with seven adipokines enhanced by at least 50% under obesity-associated metabolic conditions compared with normal physiological conditions (Figure 4B). Among the top modulated adipokines were inflammatory mediators (C-reactive protein, endocan, EN-RAGE), extracellular matrix regulating factors (Serpin A8, TIMP-1, TIMP-3), and resistin, an adipokine proposed to be an important link between obesity and diabetes. Resistin was enhanced by 48% (pre-T2D), 66% (overt T2D) and 43% (late T2D), respectively, compared with normal physiological conditions (Figure 4B).

### *CAP1* is differentially expressed across breast cancer cell lines

**Figure 5:**
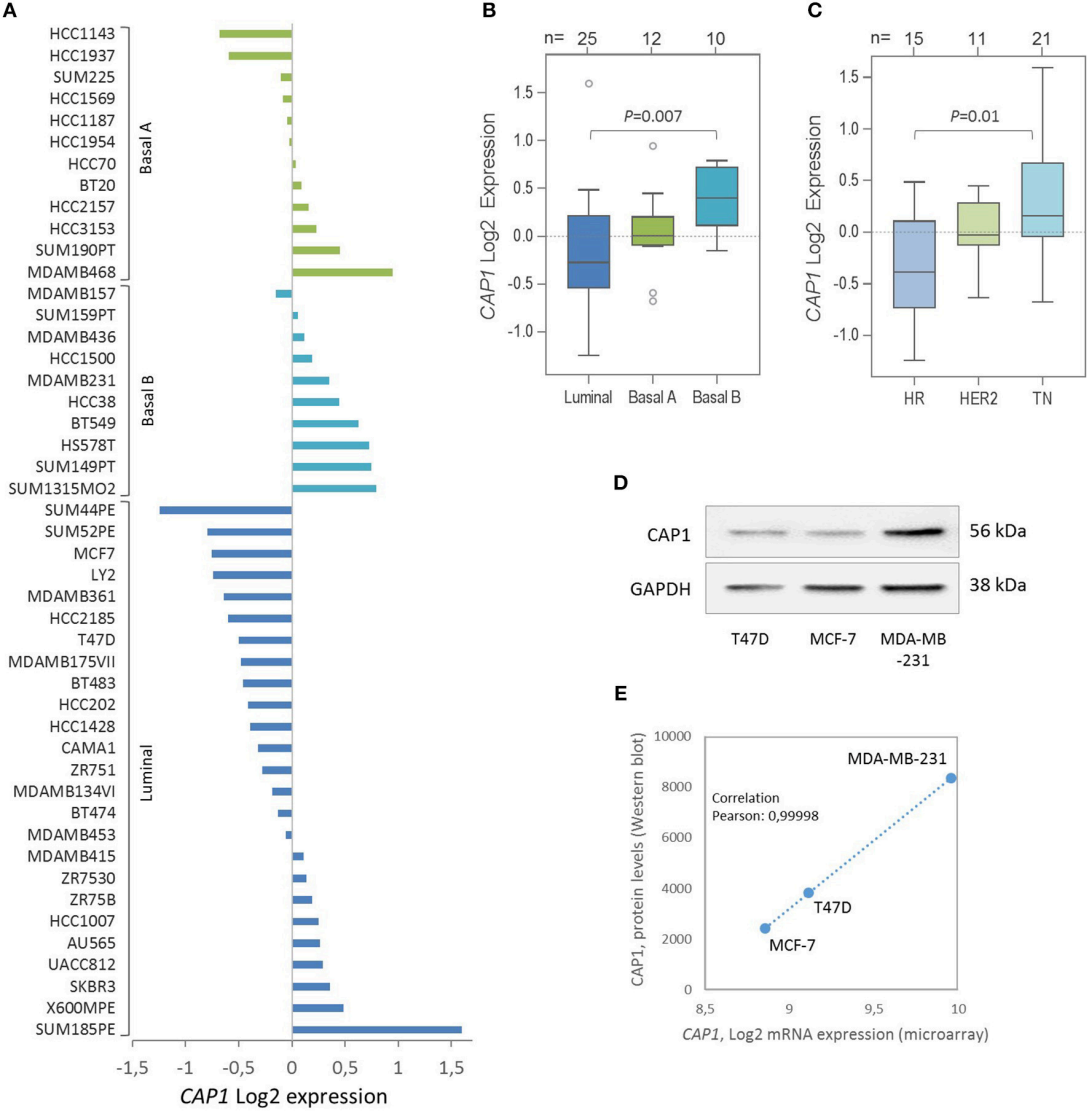
Expression of the resistin receptor CAP1 in a panel of 47 breast cancer cell lines. **(A)** *CAP1* gene expression across the 47 individual breast cancer cell lines, grouped as Basal A (green), Basal B (turquoise) and Luminal (blue) subtypes and shown as the relative *CAP1* gene expression (Log2) across the 47 cell lines using the GOBO platform. **(B**, **C)** Boxplots of *CAP1* gene expression across cell lines grouped according to **(B)** breast cancer subtypes or **(C)** hormone receptor status; hormone receptor positive (HR), HER2-positive (HER2), and triple-negative (TN), as defined by Neve et al. (22). *P*-values calculated by Mann Whitney-U test with Bonferroni correction. **(D)** CAP1 protein abundance in T47D, MCF-7 and MDA-MB-231 cells. **(E)** Graph demonstrate a positive correlation between *CAP1* mRNA expression and protein levels in T47D, MCF-7 and MDA-MB-231 breast cancer cells.

Given the recent discovery that resistin binds and acts through the CAP1 receptor, the relative *CAP1* gene expression levels were further explored across a panel of 47 breast cancer cell lines. Stratification according to breast cancer clinical subtypes (22) showed that the *CAP1* mRNA expression (Log2) was highest in the basal B subgroup with more mesenchymal-like cell lines, followed by the basal A and lowest among the luminal-like cell lines (*P* = 0.007; Figures 5A,B). Furthermore, the highest *CAP1* expression was found among triple-negative breast cancer cell lines, and the lowest among hormone receptor positive cell lines (*P* = 0.01; Figure 5C). Additional exploration of the T47D, MCF-7, and MDA-MB-231 cells showed higher CAP1 protein levels in triple-negative MDA-MB-231 cells compared with luminal-like (ER^+^) T47D and MCF-7 cells (Figure 5D). A corresponding strong positive correlation between *CAP1* mRNA expression and protein levels were found (Pearson's *r* = 0.99998; Figure 5E). These results demonstrate *CAP1* expression with subtype-specific differences across a large panel breast cancer cell lines.

### *CAP1* expression correlates with tumor subtypes and biological pathways in breast cancer patients

**Figure 6:**
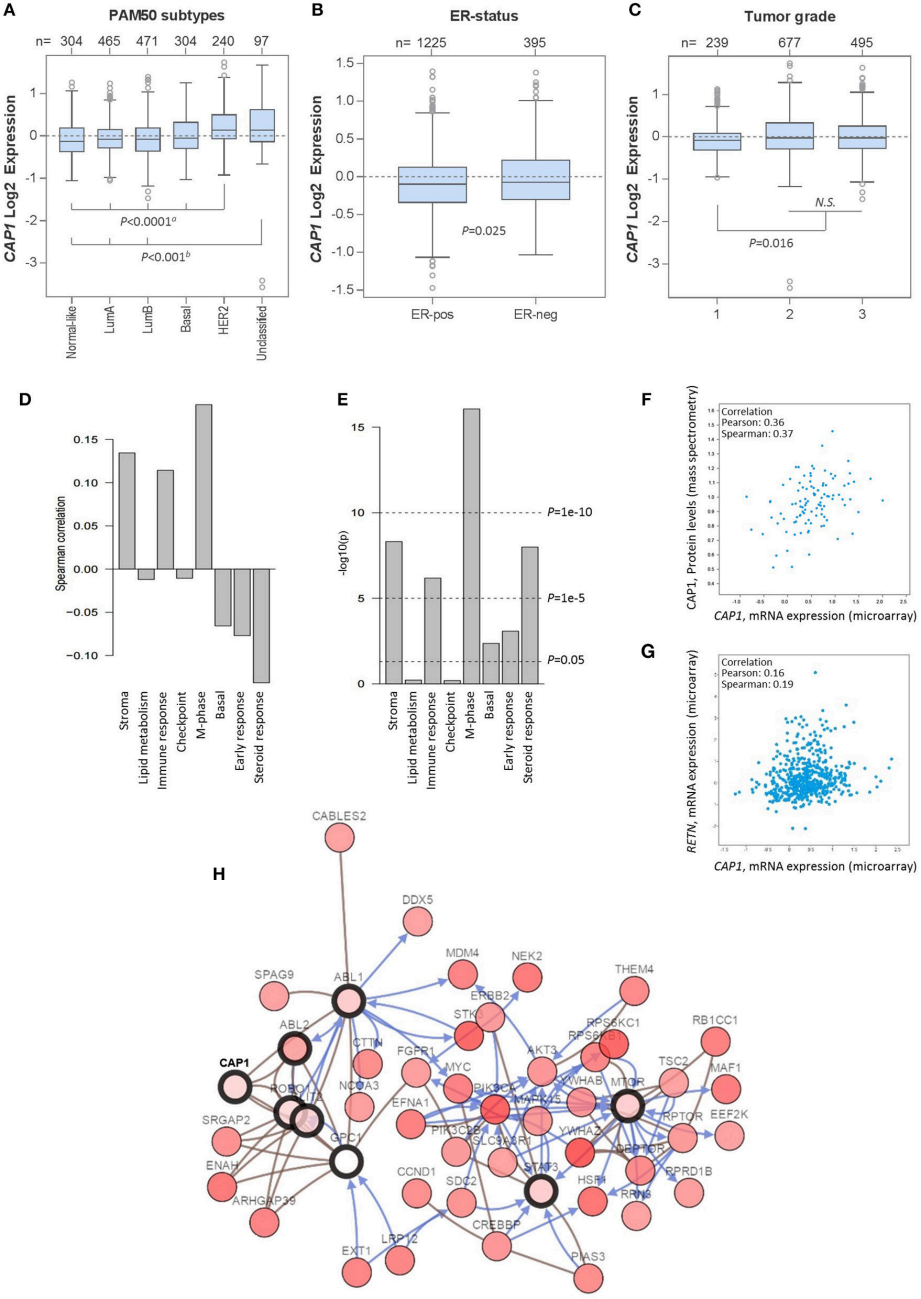
*CAP1* mRNA expression across primary breast tumors. Box plots showing *CAP1* gene expression among all breast tumors within the 1,881-sample GOBO breast cancer data set stratified according to **(A)** PAM50 subtypes, with *P*-values calculated by one-way ANOVA with Tukey's *post-hoc* test, **(B)** ER-status, with *P*-values calculated by Welch's *t*-test, and **(C)** tumor grade, with *P*-values calculated by Kruskal–Wallis test. **(D)** Correlation of *CAP1* to 8 gene modules **(E)** correlation *P*-values of *CAP1* to the 8 gene modules. Independent analyses of the 1,105-sample TCGA breast cancer data set demonstrate **(F)** positive correlation between *CAP1* mRNA expression and protein levels, and **(G)** correlation of tumor *RETN* and *CAP1* mRNA expression. **(H)** Network of CAP1 biological pathways containing 58 nodes with 8 query genes and the 50 most frequently altered neighbor genes (out of total 741). Lines with arrows (blue) indicate interactions that controls state change of gene, and connecting lines (brown) show genes in complex.

Having shown the subtype-specific *CAP1* expression in breast cancer cell lines, we sought to translationally link the experimental findings to primary human tumors by exploring gene expression data from 1,881 breast cancer patients using the GOBO platform. Similar to the expression in cell lines, *CAP1* was expressed across breast cancer subtypes (Figure 6A), with higher expression found among ER^−^ relative to ER^+^ tumors (*P* = 0.025; Figure 6B), as well among tumor grades 2 and 3 relative to grade 1 (*P* = 0.016; Figure 6C). *CAP1* expression was positively, although weakly, correlated with three gene modules associated with stroma, immune response and M-phase, while weakly negatively correlated with gene module associated with steroid response (all *P*s < 0.00001; Figures 6D,E). Separate analyzes using the independent TCGA 1,105-sample breast cancer data set showed a positive correlation between the *CAP1* mRNA expression and protein levels (Pearson's *r* = 0.36; Figure 6F), while only a weak positive correlation between *CAP1* and resistin (*RETN*) tumor expression (Pearson's *r* = 0.16; Figure 6G). Additional exploration of the top co-expressed genes with *CAP1* revealed the highest expression correlation for *RRAGC*, a crucial part in the cellular localization and activation of the mTOR complex (Supplementary Table 1). Among the top co-expressed genes were also several genes involved in actin rearrangements, cell attachment and extra cellular matrix regulation. The top 50 genes with the highest expression correlation with *CAP1* are provided as Supplementary Table 1. Network interaction analysis of *CAP1* biological pathways demonstrated *CAP1* in complex with *ABL1, ABL2, GPC1, SLIT2*, and *ROBO1*, which in turn involved further extended interactions with *FGFR1, MYC, PIK3CA, STAT3*, and *MTOR*, among others (Figure 6H). Altogether, these data demonstrate the differential expression of *CAP1* across breast cancer subtypes and imply the role of *CAP1* in both proliferative and cell motility processes.

### *CAP1* tumor expression is associated with breast cancer outcomes

**Figure 7:**
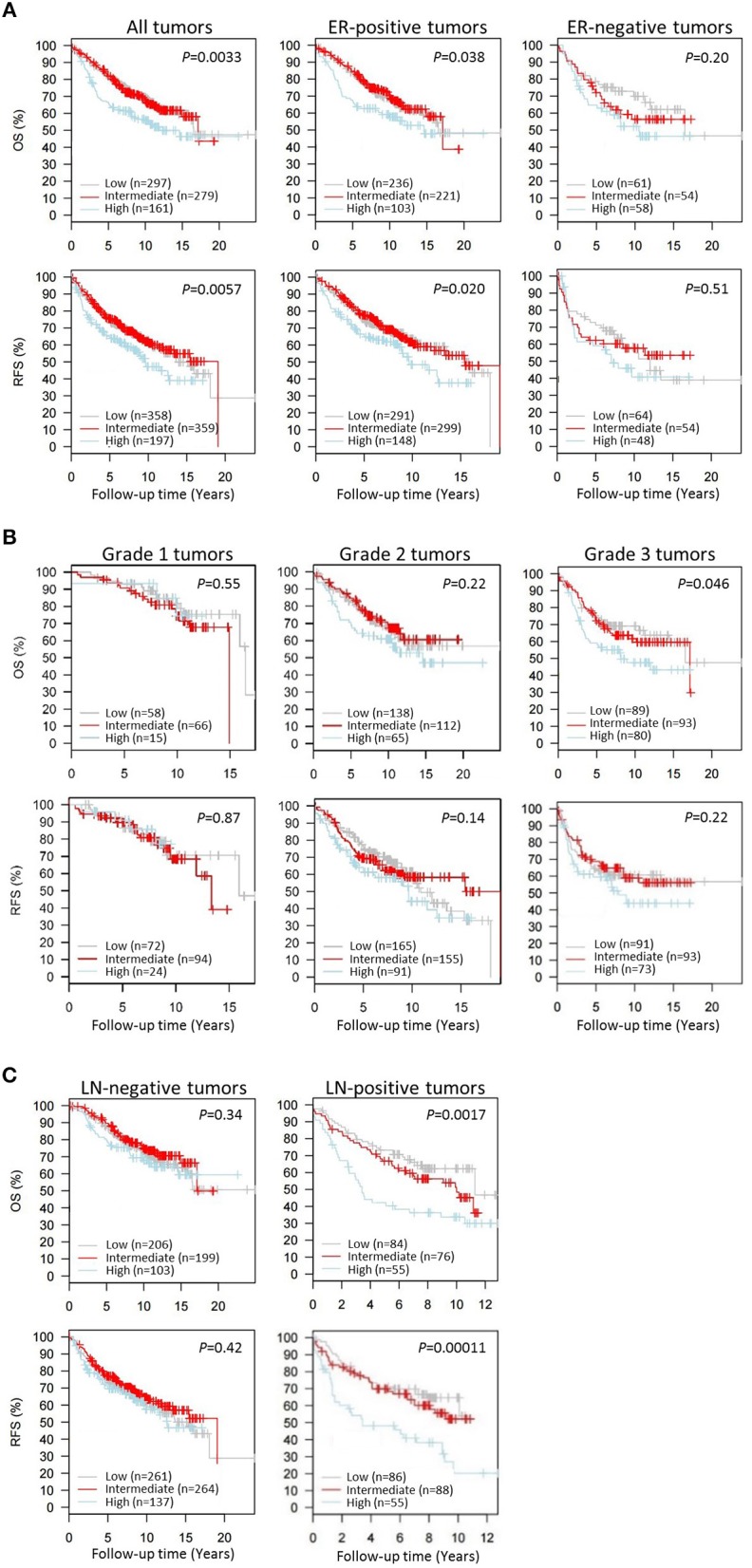
Kaplan-Meier survival analyses of the resistin receptor *CAP1* expression in relation to overall survival (OS) or relapse-free survival (RFS) in the 1,881-sample GOBO breast cancer data set. Graphs show associations of *CAP1*, categorized into tertiles based on the *CAP1* gene expression levels low, intermediate or high, with breast cancer outcomes. Percent OS or RFS **(A)** among all patients or according to tumor ER-status (positive/negative), and in relation to **(B)** tumor grade 1–3 or **(C)** lymph node involvement (LN-negative/positive) among all patients. Log-rank (2 df) *P*-values are shown, and number of patients in each tertile are indicated.

Next we wanted to evaluate the impact of *CAP1* tumor expression on breast cancer outcomes. The prognostic significance of *CAP1*, categorized into tertiles based on the *CAP1* log2 gene expression levels, and breast cancer outcomes were evaluated. High *CAP1* expression was associated with significantly shorter overall survival (OS) and relapse-free survival (RFS) among all tumors, compared with low or intermediate *CAP1* expression (Log rank; *P* = 0.003 and 0.006, respectively; Figure 7A). Stratification according to tumor ER status showed that the associations between high *CAP1* and breast cancer outcomes were most pronounced among ER-positive tumors (Log rank; *P* ≤ 0.038; Figure 7A). However, a similar non-significant trend was observed among ER-negative tumors, although with considerable fewer patients included (Figure 7A). High *CAP1* expression was additionally associated with impaired OS among patients with histologic grade 3 tumors (Log rank; *P* = 0.046), but not grade 1 or 2 tumors (Figure 7B), and with poor OS and RFS among patients with lymph node (LN-) positive tumors (Log rank; *P* ≤ 0.002; Figure 7C). The prognostic value of *CAP1* expression for breast cancer outcomes remained significant among all tumors (OS: HR_adj_ 1.54; 95% CI, 1.11–2.13 and RFS: HR_adj_ 1.47; 95% CI, 1.10–1.96), ER-positive tumors (OS: HR_adj_ 1.69; 95% CI, 1.14–2.50 and RFS: HR_adj_ 1.52; 95% CI, 1.08–2.13), and LN-positive tumors (OS: HR_adj_ 2.00; 95% CI, 1.18–3.45 and RFS: HR_adj_ 2.56; 95% CI, 1.49–4.35) after multivariable analyses adjusted for known confounders (Table 1).

**Table 1:** Multivariable Cox proportional hazard analyses for overall survival and relapse-free survival among all patients or patients with ER-positive or lymph node (LN-) positive tumors.

	**All tumors**				**ER-positive tumors**				**LN-positive tumors**			
	**Overall survival (*****n*** = **698)**		**Relapse-free survival (*****n*** = **829)**		**Overall survival (*****n*** = **533)**		**Relapse-free survival (*****n*** = **671)**		**Overall survival (*****n*** = **206)**		**Relapse-free survival (*****n*** = **217)**	
**Risk factor**	**HR^a^ (95% CI)**	***P*_adjusted_**	**HR^a^ (95% CI)**	***P*_adjusted_**	**HR^a^ (95% CI)**	***P*_adjusted_**	**HR^a^ (95% CI)**	***P*_adjusted_**	**HR^a^ (95% CI)**	***P*_adjusted_**	**HR^a^ (95% CI)**	***P*_adjusted_**
**AGE AT DIAGNOSIS**												
≤50 years	1.00 (ref)		1.00 (ref)		1.00 (ref)		1.00 (ref)		1.00 (ref)		1.00 (ref)
>50 years	1.55 (1.17–2.04)	0.002	0.79 (0.62–1.02)	0.066	1.69 (1.21–2.36)	0.002	0.78 (0.59–1.04)	0.086	1.29 (0.82–2.03)	0.268	0.61 (0.36–1.04)	0.068
**TUMOR SIZE**												
≤20 cm	1.00 (ref)		1.00 (ref)		1.00 (ref)		1.00 (ref)		1.00 (ref)		1.00 (ref)
>20 cm	1.82 (1.39–2.38)	1.44 × 10^−5^	1.76 (1.39–2.23)	2.60 × 10^−6^	1.92 (1.4–2.64)	5.87 × 10^−5^	2.14 (1.63–2.8)	4.22 × 10^−8^	2.79 (1.68–4.66)	8.22 × 10^−5^	2.15 (1.3–3.55)	2.84 × 10^−3^
**LYMPH NODE STATUS**												
Negative (0)	1.00 (ref)		1.00 (ref)		1.00 (ref)		1.00 (ref)		n.a.	n.a.	n.a.	n.a.
Positive (≥1)	2.13 (1.64–2.86)	4.69 × 10^−8^	1.49 (1.14–1.92)	0.004	2.13 (1.52–2.94)	7.60 × 10^−6^	1.41 (1.05–1.89)	0.020	n.a.	n.a.	n.a.	n.a.
**HISTOLOGIC GRADE**												
1–2	1.00 (ref)		1.00 (ref)		1.00 (ref)		1.00 (ref)		1.00 (ref)		1.00 (ref)
3	1.28 (0.94–1.73)	0.114	0.96 (0.73–1.26)	0.759	1.44 (1.02–2.03)	0.040	1.12 (0.82–1.52)	0.473	1.05 (0.67–1.65)	0.830	0.75 (0.45–1.23)	0.247
**HORMONE RECEPTOR STATUS**												
ER^+^	1.00 (ref)		1.00 (ref)		n.a.	n.a.	n.a.	n.a.	1.00 (ref)		1.00 (ref)
ER^−^	1.10 (0.80–1.52)	0.550	1.25 (0.92–1.69)	0.156	n.a.	n.a.	n.a.	n.a.	1.16 (0.71–1.92)	0.540	1.39 (0.72–2.63)	0.330
**CAP1 TUMOR EXPRESSION**												
Low	1.00 (ref)		1.00 (ref)		1.00 (ref)		1.00 (ref)		1.00 (ref)		1.00 (ref)
Intermediate	1.41 (1.01–1.92)	0.043	1.41 (1.05–1.89)	0.020	1.52 (1.03–2.22)	0.036	1.41 (1.02–1.96)	0.039	1.45 (0.88–2.38)	0.140	2.00 (1.20–4.35)	0.007
High	1.54 (1.11–2.13)	0.009	1.47 (1.10–1.96)	0.009	1.69 (1.14–2.50)	0.009	1.52 (1.08–2.13)	0.016	2.00 (1.18–3.45)	0.010	2.56 (1.49–4.35)	0.001

a *Hazard ratio (HR) adjusted for age, tumor size, lymph node involvement, histologic grade, and ER-status*.

## Discussion

The underlying biological explanations linking obesity and metabolic complications to increased risk of breast cancer and impaired prognosis have been incompletely understood (3, 28). The present study demonstrates the combined influence by adipocytes and obesity-associated metabolic conditions in promoting breast cancer cell proliferation, along with cellular alterations and induction of more motile phenotypes among both ER-positive (luminal-like) and triple-negative (basal-like) breast cancer cells. We further identified resistin among the top modulated adipokines secreted by 3T3-L1 adipocytes under obesity-associated metabolic conditions, thus constituting a plausible soluble mediator in the link between obesity, metabolic complications and breast cancer. Finally, the resistin receptor CAP1 was shown for the first time in the present study to be expressed across a large panel of breast cancer cell lines and primary human tumors, and that high CAP1 expression was associated with poor tumor characteristics and impaired prognosis among breast cancer patients.

The majority of previous studies have focused on effects by adipocytes alone on breast cancer, without the context of metabolic alterations associated with obesity. Herein, we developed *in vitro* models mimicking an obesity-associated metabolic state. The present findings show that ER^+^ (T47D and MCF-7) and most strongly triple-negative MDA-MB-231 breast cancer cells display enhanced proliferation in the presence of soluble adipocyte-derived factors. Interestingly, under insulin resistance (overt T2D) or impaired insulin secretion (late T2D)-associated conditions, the growth promoting actions by adipocytes were further increased. Also obesity-associated metabolic conditions, in the absence of adipocytes, stimulated the proliferation of MCF-7 and MDA-MB-231 cells in part. These results suggest that obesity-associated metabolic alterations may promote breast cancer cell proliferation *per se*, as well as stimulate the release or sensitivity to adipocyte-derived soluble mediators. In line with our findings, a previous report showed that co-culture with human adipocytes promoted breast cancer cell viability, which was further modulated by hyperglycemia and fatty acids via the induction of adipocyte-derived IGF1 and interleukin-8 (29).

A large study of 53,816 Danish breast cancer patients reports that overweight and obese women presented with more aggressive tumor characteristics at diagnosis and had an increased risk of distant metastases and breast cancer-related death, compared with patients of normal weight (8). In line with this, the present findings show that breast cancer cells cultured in the presence of the adipocyte secretome may exhibit cellular adaptations indicative of more motile features. Contrary to the growth promoting responses, the low proliferative and epithelial-like T47D cells demonstrated the most pronounced morphological alterations with increased abundance of stellate protrusion and activation of motility in response to adipocytes and obesity-associated metabolic conditions, followed by the more proliferative MCF-7 or triple-negative MDA-MB-231 cells. Although our models showed the most pronounced induction of migration in the two low-invasive ER^+^ cells, others have demonstrated a significant role of the adipose microenvironment in facilitating also triple-negative breast cancer cell motility and invasiveness (30–32). Co-culture with 3T3-L1 adipocytes may induce a similar yet a more marked response in breast cancer cells compared to that induced by adipocyte secretome alone, due to the continuous exchange of soluble mediators (32). This may in part explain the difference in magnitude of response between studies.

When exposed to obesity-associated metabolic conditions, we show that adipocytes increase the secretion of resistin, together with inflammatory mediators (C-reactive protein, endocan and EN-RAGE) and extracellular matrix regulating factors (Serpin A8, TIMP-1, and TIMP-3), which may contribute to the biological effects observed. Circulating resistin levels are positively correlated with both visceral and subcutaneous adipose depots in women, and are higher in patients with type 2 diabetes than among individuals without diabetes (33, 34). Furthermore, resistin has been reported to be elevated in obese individuals and among breast cancer patients (not adjusted for BMI), although the results have been conflicting (11–14). While several studies have shown increased systemic and breast tumor resistin levels among postmenopausal breast cancer patients to be positively associated with increasing BMI, poor tumor characteristics and impaired prognosis, an inverse association was recently reported among early onset breast cancer patients, independent of BMI (13, 35, 36). In this cohort of chemotherapy-treated patients, aged 40 years or younger, higher circulating resistin levels were in contrast associated with lymph-node negative disease and longer disease-free survival (36). The disparate resistin effects in different patient populations are potentially highly interesting and may in part contribute to the crossover effects observed in epidemiology studies demonstrating a positive association between increasing BMI and postmenopausal breast cancer risk, while a suggested risk reduction among young adults (3, 37).

Although current data on the biological role of resistin in breast cancer is very limited, it has been found to promote breast cancer progression via stimulation of proliferation, migration and metastatic behavior of breast cancer cells through interleukin-6, STAT-3 and c-Src activation, along with induction of epithelial-mesenchymal transition and the mesenchymal marker vimentin (38–40). Resistin has further been demonstrated to facilitate chemoresistance and attenuate doxorubicin-induced apoptosis in breast cancer cells (41). Adipose-tissue secreted resistin in mice is primarily derived from mature adipocytes and impact on metabolic regulation. In humans, resistin expression is also found in non-adipocyte cells such as monocytes and macrophages, suggesting additional inflammatory properties of resistin (42). The effects by resistin has been suggested to be mediated via TLR4-induced NF-κB activation and more recently through interactions with CAP1 (15, 40, 43, 44). The recent discovery of the novel resistin receptor CAP1 will facilitate further exploration of the resistin-CAP1 link in obesity-associated breast cancer.

CAP1 interacts with actin filaments and can regulate cytoskeletal dynamics and cell motility (15, 45). In breast cancer, CAP1 has been suggested to negatively regulate E-cadherin expression, thus reducing cell adhesion and promote migration (46). Similarly, CAP1 knock down reduced both breast cancer cell proliferation and migration (47). However, a separate study suggested that CAP1 exert distinct functions dependent on breast cancer subtype, as CAP1 depletion in highly invasive MDA-MB-231 cells stimulated proliferation and migration, contrary to ER^+^ MCF-7 cells where CAP1 knock down inhibited migration (48). The conflicting literature reports highlight the importance of further studies to map the role of CAP1 in breast cancer progression. Herein, we show for the first time that *CAP1* is expressed across a large panel of breast cancer cell lines and primary human tumors. Higher *CAP1* expression was found among ER^−^ breast tumors, while lower expression was found among ER^+^ tumors. Yet, high *CAP1* expression among breast cancer patients with ER^+^ or lymph node positive tumors was associated with significantly shorter overall and relapse-free survival, compared with low or intermediate expression. Furthermore, high *CAP1* expression was associated with poor tumor characteristics and correlated with expression of genes and biological pathways within growth promoting (such as Abl, RRAGC and mTOR) and cell motility processes (e.g., Arp2/3 complex, ARSB and FN1) in breast cancer.

It was recently reported that certain resistin (*RETN)* and *CAP1* gene variants were associated with increased risk of breast cancer among Mexican women (13). While no association between the *RETN* polymorphism and serum- or tumor resistin expression was found, it was shown to correlate with *CAP1* tumor expression and breast cancer metastasis (13). In the present study, only a modest positive correlation between *RETN* and *CAP1* tumor expression was found. However, the tumor resistin expression will not reflect the accumulated exposure to circulating resistin levels over time, for which additional analyses in serum samples would be required. Considering the independent prognostic role of CAP1 shown in the present study and the importance of CAP1-related biological processes in breast cancer (49–52), further investigations are needed to elucidate the clinical implication of the resistin-CAP1 link in obesity-associated breast cancer.

Taken together, the published evidence in various models and the present results supports the hypothesis that adipocytes and obesity-associated metabolic conditions may jointly influence breast cancer cell progression. The present study utilized the well-established and most extensively used adipocyte model (3T3-L1) for the study of obesity-related characteristics. Additional exploration of the crosstalk between human adipose tissue and breast cancer with validation in independent population-based cohorts are warranted to recapitulate the characteristics of the human disease. The present data suggest that 3T3-L1 adipocyte-derived mediators, including resistin, significantly stimulate the proliferation of both luminal A-like and triple-negative human breast cancer cells along with the induction of cellular adaptations linked to enhanced motility, which are further enhanced under obesity-associated metabolic conditions. Further insights into the biological mechanisms and obesity-associated mediators involved in the link between obesity and breast cancer, may open for improved patient information and potential new strategies to prevent breast cancer and optimize treatment of the disease.

## Author contributions

AR and SB conceived the study, oversaw the project and provided funding. AR and MB performed the experiments, acquired and analyzed the data. BL assisted with lipid staining experiments. SK consulted on gene expression data analyses. All authors contributed to review and/or revision of the manuscript, and approved the final manuscript.

### Conflict of interest statement

SB receives consultant fees from Roche and Novartis. The remaining authors declare that the research was conducted in the absence of any commercial or financial relationships that could be construed as a potential conflict of interest.

## Abbreviations

CAP1: Adenylate Cyclase-Associated Protein 1
T2D: type 2 diabetes
IGF-I: Insulin-like Growth Factor I
ER: Estrogen receptor
Adipo-CM: Adipocyte conditioned media
HR: Hazard Ratio
OS: Overall Survival
RFS: Relapse-Free Survival
LN: Lymph node.
