# Supplementary material for: Adipocytes and Obesity-Related Conditions Jointly Promote Breast Cancer Cell Growth and Motility: Associations With CAP1 for Prognosis
Source: Front Endocrinol (Lausanne). 2018 Nov 22;9:689. doi: 10.3389/fendo.2018.00689 (PMC6262006; doi:10.3389/fendo.2018.00689)
Supplement: Supplementary file 1 [file Table_1.DOCX]

Supplementary Material

Adipocytes and obesity-related conditions jointly impact on breast cancer: associations with CAP1

Ann H. Rosendahl*, Malin Bergqvist, Barbara Lettiero, Siker Kimbung, Signe Borgquist

*** Correspondence:** Associate Professor Ann Rosendahl: ann.rosendahl@med.lu.se

**Supplementary Table S1.** Table of the top 50 genes with the highest expression correlation with *CAP1*

|  | **Gene Symbol** |  | **Cyto-band** |  | **Pearson Score** |  | **Spearman Score** |  | **Description** |
| --- | --- | --- | --- | --- | --- | --- | --- | --- | --- |
|  | **RRAGC** |  | 1p34 |  | 0.51 |  | 0.54 |  | Ras Related GTP Binding C |
|  | **ACTR3** |  | 2q14.1 |  | 0.49 |  | 0.58 |  | ARP3 Actin Related Protein 3 Homolog |
|  | **ARSB** |  | 5q14.1 |  | 0.47 |  | 0.55 |  | Arylsulfatase B |
|  | **ZMPSTE24** |  | 1p34 |  | 0.46 |  | 0.37 |  | Zink Metallopeptidase STE24 |
|  | **FN1** |  | 2q34 |  | 0.45 |  | 0.52 |  | Fibronectin 1 |
|  | **RLF** |  | 1p32 |  | 0.45 |  | 0.44 |  | Rearranged L-Myc Fusion |
|  | **ARPC2** |  | 2q36.1 |  | 0.45 |  | 0.55 |  | Actin Related Protein 2/3 Complex Subunit 2 |
|  | **ACTR2** |  | 2p14 |  | 0.44 |  | 0.51 |  | ARP2 Actin-Related Protein 2 Homolog |
|  | **ACBD4** |  | 17q21.31 |  | -0.43 |  | -0.48 |  | Acyl-CoA Binding Domain Containing 4 |
|  | **CAPZA1** |  | 1p13.2 |  | 0.43 |  | 0.49 |  | Capping Actin Protein of Muscle Z-line Alpha Subunit 1 |
|  | **ITGB1** |  | 10p11.2 |  | 0.43 |  | 0.48 |  | Integrin Subunit Beta 1 |
|  | **ITGB6** |  | 2q24.2 |  | 0.43 |  | 0.35 |  | Integrin Subunit Beta 6 |
|  | **AKIRIN1** |  | 1p34.3 |  | 0.43 |  | 0.44 |  | Akirin 1 |
|  | **INHBA** |  | 7p15-p13 |  | 0.42 |  | 0.48 |  | Inhibin Beta A Subunit |
|  | **PLAU** |  | 10q22.2 |  | 0.42 |  | 0.5 |  | Plasminogen Activator, Urokinase |
|  | **ANTXR1** |  | 2p13.1 |  | 0.42 |  | 0.47 |  | Anthrax Toxin Receptor 1 |
|  | **HRH1** |  | 3p25 |  | 0.41 |  | 0.48 |  | Histamine Receptor H1 |
|  | **WNT2** |  | 7q31.2 |  | 0.41 |  | 0.47 |  | Wnt Family Member 2 |
|  | **FAM26E** |  | 6q22.1 |  | 0.41 |  | 0.48 |  | Family With Sequence Similarity 26 Member E |
|  | **MFSD2A** |  | 1p34.2 |  | 0.41 |  | 0.4 |  | Major Facilitator Superfamily Domain Containing 2A |
|  | **KAT2A** |  | 17q21 |  | -0.4 |  | -0.5 |  | Lycin Acetyltransferase 2A |
|  | **LINC00663** |  | 19p13.11 |  | -0.4 |  | -0.44 |  | Long Intergenic Non-Protein Coding RNA 663 |
|  | **LOX** |  | 5q23.2 |  | 0.4 |  | 0.49 |  | Lysyl Oxidase |
|  | **CORO1C** |  | 12q24.1 |  | 0.4 |  | 0.5 |  | Coronin 1C |
|  | **FUZ** |  | 19q13.33 |  | -0.39 |  | -0.45 |  | Fuzzy Planar Cell Polarity Protein |
|  | **ACVR1** |  | 2q23-q24 |  | 0.39 |  | 0.43 |  | Activin A Receptor Type 1 |
|  | **FBN1** |  | 15q21.1 |  | 0.39 |  | 0.47 |  | Fibrillin 1 |
|  | **FAP** |  | 2q23 |  | 0.39 |  | 0.46 |  | Fibroblast Activation Protein Alpha |
|  | **HIVEP2** |  | 6q23-q24 |  | 0.39 |  | 0.42 |  | Human Immunodeficiency Virus Type I Enhancer Binding Protein 2 |
|  | **LIMS1** |  | 2q12.3 |  | 0.39 |  | 0.46 |  | LIM Zink Finger Domain Containing 1 |
|  | **IQGAP1** |  | 15q26.1 |  | 0.39 |  | 0.45 |  | IQ Motif Containing GTPase Activating Protein 1 |
|  | **PLXNC1** |  | 12q23.3 |  | 0.39 |  | 0.5 |  | Plexin C1 |
|  | **LHFPL2** |  | 5q14.1 |  | 0.39 |  | 0.49 |  | Lipoma HMGIC Fusion Partner-Like 2 |
|  | **ADAMTS2** |  | 5qter |  | 0.39 |  | 0.48 |  | ADAM Metallopeptidase With Trombospondin Type 1 Motif 2 |
|  | **TRAM2** |  | 6p21.1-p12 |  | 0.39 |  | 0.46 |  | Translocation Associated Membrane Protein 2 |
|  | **SH3GLB1** |  | 1p22 |  | 0.39 |  | 0.49 |  | SH3 Domain Containing GRB2 Like Endophilin B1 |
|  | **CEMIP** |  | 15q24 |  | 0.39 |  | 0.41 |  | Cell Migration Inducing Hyaluronan Binding Protein |
|  | **ENTPD7** |  |  |  | 0.39 |  | 0.41 |  | Ectonucleoside Triphosphate Diphosphohydrolase 7 |
|  | **NID2** |  | 14q22.1 |  | 0.39 |  | 0.46 |  | Nidogen 2 |
|  | **CCNYL1** |  | 2q33.3 |  | 0.39 |  | 0.49 |  | Cyclin Y Like 1 |
|  | **RNF11** |  | 1p32 |  | 0.39 |  | 0.37 |  | Ring Finger Protein 11 |
|  | **NECAB3** |  | 20q11.22 |  | -0.38 |  | -0.48 |  | N-Terminal EF-Hand Calcium Binding Protein 3 |
|  | **BACH1** |  | 21q22.11 |  | 0.38 |  | 0.47 |  | BTB Domain AND CNC Homolog 1 |
|  | **CALD1** |  | 7q33 |  | 0.38 |  | 0.46 |  | Caldesmon 1 |
|  | **CALU** |  | 7q32.1 |  | 0.38 |  | 0.5 |  | Calumenin |
|  | **KPNA3** |  | 13q14.3 |  | 0.38 |  | 0.43 |  | Karyopherin Subunit Alpha 3 |
|  | **PSMD5** |  | 9q33.2 |  | 0.38 |  | 0.35 |  | Proteasome 26S Subunit, Non-ATPase 5 |
|  | **LOC284441** |  | 19p12 |  | 0.38 |  | 0.41 |  | Actin-Related Protein 2 Pseudogene |
|  | **ADAM19** |  | 5q33.3 |  | 0.38 |  | 0.55 |  | ADAM Metallopeptidase Domain 19 |
|  | **PICALM** |  | 11q14 |  | 0.38 |  | 0.48 |  | Phosphatidylinositol Binding Clathrin Assembly Protein |
